# Regulation of the actin cytoskeleton in *Helicobacter pylori*-induced migration and invasive growth of gastric epithelial cells

**DOI:** 10.1186/1478-811X-9-27

**Published:** 2011-11-01

**Authors:** Silja Wessler, Mario Gimona, Gabriele Rieder

**Affiliations:** 1 Division of Molecular Biology, Department of Microbiology, University of Salzburg, Salzburg, Austria; 2 University Clinic for Blood Group Serology and Transfusion Medicine, PMU, General Hospital Salzburg, SALK, Salzburg, Austria

**Keywords:** *Helicobacter pylori*, type IV secretion system, CagA, actin cytoskeleton

## Abstract

Dynamic rearrangement of the actin cytoskeleton is a significant hallmark of *Helicobacter pylori *(*H. pylori*) infected gastric epithelial cells leading to cell migration and invasive growth. Considering the cellular mechanisms, the type IV secretion system (T4SS) and the effector protein cytotoxin-associated gene A (CagA) of *H. pylori *are well-studied initiators of distinct signal transduction pathways in host cells targeting kinases, adaptor proteins, GTPases, actin binding and other proteins involved in the regulation of the actin lattice. In this review, we summarize recent findings of how *H. pylori *functionally interacts with the complex signaling network that controls the actin cytoskeleton of motile and invasive gastric epithelial cells.

## Review

**Figure 1:**
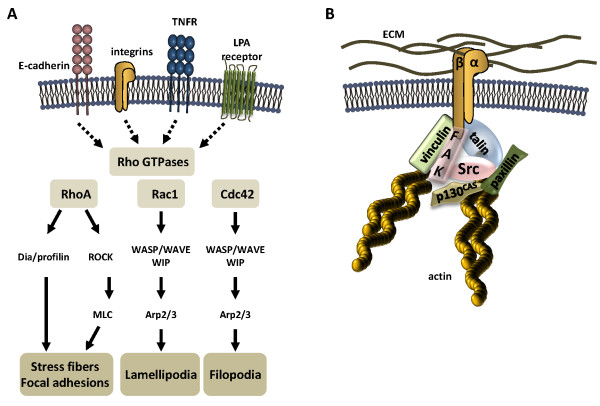
**Signal transduction pathways involved in the regulation of the actin cytoskeleton**. **(A) **The formation of actin-dependent structures, such as stress fibers, focal adhesions, lamellipodia, and filopodia is controlled by cell surface molecules ranging from E-cadherin and integrins to receptors for small components (*e.g*. TNF-α or LPA) allowing the transmission of extracellular stimuli to the actin cytoskeleton. The Rho GTPases RhoA, Rac1, and Cdc42 are key elements in the regulation of actin filaments. Rac1 and Cdc42 induce actin polymerization through WASP/WAVE family members and WIPs stimulating the Arp2/3 complex. RhoA regulates Dia1/profilin and the ROCK/MLC pathways to promote polymerization of F-actin. **(B) **Focal adhesions are important structures in linking the ECM to the intracellular actin cytoskeleton via α and β integrin heterodimers. The extracellular part of integrins binds to proteins of the ECM, while the intracellular domain recruits a wide range of intracellular signaling (FAK, Src, *etc*.) and adaptor proteins (talin, paxillin, vinculin, or p130^CAS^, *etc*.) to connect the actin cytoskeleton.

The continuous reorganization and turnover of the actin cytoskeleton is a fundamental process in the regulation of cell adhesion to neighboring cells and extracellular matrix (ECM), phagocytosis, cell shape or migration. Generally, actin exists in cells as monomeric globular actin (G-actin) and filamentous actin (F-actin), which are formed upon polymerization of G-actin monomers in a defined directionality. A wide range of upstream signaling molecules including the cell adhesion molecule E-cadherin, integrins, components of the ECM, or stimuli such as tumor necrosis factor alpha (TNF-α) and lysophosphatidic acid (LPA) are known in the transmission of extracellular signals to the actin cytoskeleton allowing rapid reactions to a changing environment (Figure 1A). Hence, remodeling of the actin cytoskeleton architecture depends on a large group of signaling molecules that bind to actin and modulate the assembly of the actin network (see [1] for a comprehensive overview).

Among actin-dependent cellular processes, efficient cell migration requires a coordinated rearrangement of the actin lattice in motile cells. Polymerization of F-actin at cell protrusions triggers the formation of sheet-like lamellipodia and rod-like filopodia pushing migrating cells [2,3]. Additionally, formation of contractile structures through interaction of actin with myosin II pulls the cell body across the ECM. Those processes involve a wide range of actin binding proteins (e.g. cortactin, α-actinin, fascin, profilin, filamin, *etc*.) that contribute to actin stabilization, bundling and branching, forming a complex network. Signaling pathways modulating actin rearrangement are complex and have been covered in several excellent reviews [4-6]. Summarizing the most important findings in a simplistic model (Figure 1A), signaling pathways initiated at cell surface receptors to promote distinct membrane protrusions converge on Rho family GTPases as the key elements of signal transduction. One of the best characterized Rho GTPase family members is RhoA regulating the formation of stress fibers and focal adhesion assembly, while Rac and Cdc42 are mainly involved in membrane ruffling and formation of filopodia, respectively [4]. Rac1 and Cdc42 can induce actin polymerization through members of the Wiskott-Aldrich syndrome protein (WASP) family and WASP-interacting proteins (WIPs). The WASP family of actin nucleation promoting factors (NPFs) includes WASP, N-WASP and four forms of WASP verprolin homologous protein (WAVE). Through a conserved C-terminal domain, WASP proteins stimulate the actin-related proteins 2/3 (Arp2/3) complex activity to nucleate actin filaments and to elongate at their free barbed ends. Stress fiber assembly and contraction are predominantly induced by RhoA [7] as mentioned above, which controls Dia1/profilin to promote polymerization of F-actin [8]. Another mechanism involves Rho-induced Rho-associated serine/threonine kinase (ROCK) as an important downstream effector to induce myosin light chain (MLC) phosphorylation [9] (Figure 1A).

Commonly, contractile stress fibers attach to the plasma membrane at nascent focal adhesions, which are stabilized by α and β integrin heterodimeric receptors (Figure 1B). Bridging the ECM to the actin cytoskeleton, the integrin ectodomain directly binds to ECM proteins (e.g. fibronectin), while the intracellular domain is connected to the actin cytoskeleton via recruited adaptor and signaling proteins including focal adhesion kinase (FAK), vinculin, talin and paxillin [6]. Upon activation, FAK recruits the non-receptor tyrosine kinase c-Src to the focal adhesion sites in order to phosphorylate other focal adhesion proteins such as paxillin and p130^Cas ^leading to mature focal adhesions (Figure 1B). The integrity and maturation of focal adhesion complexes cycle between assembly at the protrusions and disassembly at the trailing edge of migrating cells; however the precise molecular mechanisms are not completely understood. In this review, we summarize the current findings on how the human carcinogen *Helicobacter pylori *(*H. pylori*) controls the host cell actin cytoskeleton to form stress fibers and deregulates adhesion complexes to induce changes in cell shape, migration and invasive growth.

## *H. pylori *induces migration and invasive growth of gastric epithelial cells

*H. pylori *is one of the most successful human pathogen that colonizes the gastric lining epithelium in the stomach of approximately 50% of the world's population. Once acquired and not eradicated by antibiotics, *H. pylori *normally persists throughout lifespan since the host is unable to clear the infection. Only a minority of 10-15% of infected individuals develops severe gastric diseases which mainly depend on bacterial expressed pathogenic and virulence factors, environmental determinants and individual genetic predispositions (*e.g*. polymorphisms of host genes such as interleukin-1β (IL-1β), IL-8, IL-10, runt-related gene 3 (RUNX3), *etc*.), which can influence gastric atrophy and carcinogenesis [10-12]. Most severe complications are inflammatory disorders involving acute and chronic gastritis or ulceration of the stomach and duodenum, which can eventually result in Mucosa Associated Lymphoid Tissue (MALT) lymphoma and gastric cancer [13]. According to its capability to promote cancer, *H. pylori *was classified by the World Health Organization as a class-I carcinogen [14].

*H. pylori *pathogenesis is dependent on the expression of bacterial virulence factors [10], which might involve complex cellular responses of gastric epithelial cells [15,16]. The vacuolating cytotoxin A (VacA) is secreted by many, if not all, *H. pylori *isolates and might enhance the *H. pylori *virulence though its pleiotropic functions *in vivo*. VacA binds to many surface factors, including the receptor-like protein tyrosine phosphatase alpha and beta (RPTPα and RPTPβ) presented on host cells and, after uptake, induces membrane anion-selective channels and pore formation, apoptosis and gigantic vacuoles in host cells [17]. VacA is further associated with the inhibition of T-cell function through binding to the β2 integrin receptor [18,19]. Another important pathogenic factor is the cytotoxin-associated gene A (CagA), which has attracted much attention since its expression is closely associated with the development of severe diseases *in vivo *[20,21]. The *cagA *gene is located within the *cag *pathogenicity island (*cag*PAI) region on the bacterial chromosome, which encodes proteins important for structure and function of a specialized type IV secretion system (T4SS) [22,23]. Importantly, it has been demonstrated that the *cag*PAI protein CagL represents a T4SS-pilus associated adhesin for α5β1 integrin expressed on the epithelial host cell surface. Binding of the fibronectin-mimicking Arg-Gly-Asp (RGD) motif in the CagL molecule to β1 integrin is necessary to translocate CagA into the host cytoplasm [24,25]. Many studies described that CagA-positive *H. pylori *strains are closely connected with the development of acute gastritis, pre-neoplastic and neoplastic lesion [26-29]. Causative associations between CagA and the formation of neoplasia were demonstrated in Mongolian gerbils [30,31] and in a transgenic mouse model in which CagA induced neoplastic transformations *in vivo *[32].

**Figure 2:**
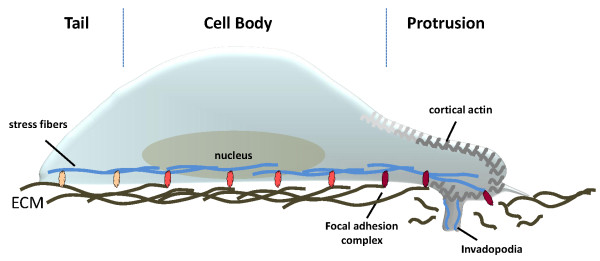
**Model of migrating epithelial cells**. For efficient migration, epithelial cells develop new actin-dependent protrusions which are connected to the ECM via newly assembled focal adhesions (red) at the leading edge. Secretion of proteases to degrade ECM is required to extend the protrusion into the ECM to form invadopodia. At the tail, matured focal adhesions (grey) disassemble to facilitate the movement of the cell body in a defined direction.

In healthy individuals, the gastric epithelium represents effective first barriers against pathogens, which is tightly sealed by coordinated regulation of epithelial cell shape, polarity, cell-to-cell and cell-to-matrix adhesions. Concomitantly with colonization of the gastric mucus, *H. pylori *dismantles the epithelial barrier function to induce inflammatory responses and neoplastic changes dependent on *H. pylori *virulence factors [33]. This might be facilitated by a rearrangement of the actin cytoskeleton as a central mechanism in those processes. Supporting this suggestion, *H. pylori *induces the formation of protrusions and massive stress fibers in cultured gastric epithelial cells accompanied by the loss of epithelial morphology and cell-to-cell adhesions leading to a mitogenic-invasive scattering phenotype *in vitro *[33,34] reminiscent of growth factor-induced Epithelial-Mesenchymal Transition (EMT). The EMT phenotype requires a complex morphogenetic program initiated by alteration of gene expression, the loss of typical epithelial properties and the increase of mesenchymal characteristics [35], which could be detected in *H. pylori*-colonized cells [36]. During EMT, cells lose their polar, epithelial nature and acquire a highly motile, mesenchymal morphology. Principally, EMT is defined by the (i) disassembly of intercellular junctions, (ii) reorganization of the actin cytoskeleton from cell-cell and cell-matrix junctions into protrusive and invasive pseudopodial structures such as actin stress fibers and actin-dependent protrusion of cell pseudopodia, (iii) and an increase of cell motility. In general, these processes occur in synchronous fashion, but independently from each other [35]. Accordingly, efficient *H. pylori*-mediated cell migration is an extremely complex coordinated process which is initiated by the extension of lamellipodia at the leading edge of the cell, assembly of new focal adhesion complexes, secretion of proteases to degrade contacts to the ECM supporting the formation of invadopodia, development of contractile forces and finally disassembly of focal adhesions leading to tail detachment (Figure 2) [34,37].

Actin-dependent protrusion of pseudopodial surface extensions is a key element during EMT-related migration of *H. pylori*-colonized cells. Pathogenic *H. pylori *strains induce a morphogenetic program in different gastric epithelial cell lines that closely resembles the features of EMT [36]. CagA-transfected cells invade through the extracellular matrix *via *the formation of invasive pseudopodia [38] indicating that CagA might induce EMT in gastric cancer cells. Functionally these structures mimic invasive podosomes or invadopodia, and show a similar dependence on matrix metalloproteases (MMPs) for invasion. In support of this concept non-invasive podosomes have been shown to become gradually replaced by invasive invadopodia in EMT (Figure 2) [39].

Adhesion based spatio-temporal orchestration of actin-polymerization-driven invasive structures [40] is a feature of many physiological and pathological events. The players in this scenario are mechano-sensitive molecules that also depend on integrin-mediated outside-in signaling cascades and involve many of the same players (such as ezrin, Abl, Src, *etc*.) that are required for *H. pylori*-induced cell invasion into neighboring tissues [38,41,42]. A significant element that separates invadopodia from focal adhesions is the modulation of the cell's secretory machinery and the focal secretion of ECM-degrading matrix metalloproteases (MMPs) that ultimately allow the breach of tissue boundaries [43]. The ultrastructural features and intracellular dynamics of *H. pylori*-induced pseudopods are still poorly defined, but a future identification of these structures as invadopodia-related cellular protrusions would not be a surprise.

As infection with *cagA*-positive strains of *H. pylori *is tightly associated with the induction of gastric adenocarcinoma the targets highjacked by injected CagA likely controls pseudopod formation and invasion of infected motile cells. Indeed, *in vitro *studies have shown that CagA binds the adapter molecule growth factor receptor bound protein 2 (Grb2) [44], which can link Abl and Src kinase signaling cascades to MMP expression and invadopodium formation [45] and can thus contribute to the site-specific formation of signaling complexes required for cell migration and invasive growth. Interestingly, *H. pylori *induces expression of MMP-7 at the lamellipodia of motile cells, which was also triggered by activated RhoA and Rac [46], suggesting a close connection between ECM degradation, invasive growth and efficient cell motility. The cortical cytoskeleton serves as a nexus between the extracellular environment and the cytoplasm, and is positioned to coordinate cellular signal relays. It comes thus as no surprise that cytoskeleton-associated cortical proteins have key roles in *H. pylori*-induced cell modulation. The mucin-like transmembrane glycoprotein podoplanin can also induce EMT, cell migration and invasive growth by recruiting the ERM (Ezrin, Radixin, Moesin)-family protein ezrin, an organizer of the cortical cytoskeleton, to the plasma membrane. This interaction is essential for the activation of the RhoA/ROCK pathway by podoplanin [47,48]. In *H. pylori*-infected gastric epithelial cells, ezrin becomes dephosphorylated which could be involved in the development and metastasis of *H. pylori-*induced gastric cancer [49]. Ezrin's dual role as an actin binding and GTPase scaffolding protein further identifies this molecular complex as a key target for understanding the cytoskeletal rearrangements that lead to migration and invasive growth of infected epithelial cells [49,50].

In fact, the EMT-like phenotype of *H. pylori*-infected epithelial host cells implies the formation of protrusions and elongation. Rather unfortunate, terms like 'scattering phenotype' or 'hummingbird phenotype' in connection with *H. pylori *infection has been widely become synonymous with 'cell elongation' or 'cell migration'. Interestingly, data are accumulating indicating that cellular elongation and motility are differentially regulated by *H. pylori *via independent signal transduction pathways [51]. Consistently observed, the drastic elongation of host cells is strictly dependent on CagA injection [52-54], while *H. pylori*-induced cell motility is *cag*PAI-dependent, but largely CagA-independent [42,51,55]. Making those observations more complex, data are accumulating that CagA and VacA functions are antagonizing each other in some assays. In accordance with a study showing that specific VacA variants inhibited CagA-dependent cell elongation, CagA reduced VacA-mediated apoptosis and *vice versa*, underscoring the interfering functions of pathogenic factors expressed by *H. pylori *[56,57]. Furthermore, *H. pylori*-expressed pathogenic factors might differentially interact with host cells leading to the disruption of the gastric epithelium and determining the outcome of gastric disorders. Rapid host cell elongation and migration are particular evident in human gastric cancer cells (e.g. AGS cells) [53,58,59], breast cancer cells (e.g. MCF-7 cells) [42,60], and a subtype of the canine kidney cell line MDCK [55,61]. Milder and less pronounced development of this typical phenotype was observed in gastric MKN-1, MKN-28 and Hs-746T cells within early phases of *H. pylori *infections [15]. In terms of cell morphological and junctional changes, only few reports on primary gastric epithelial cells are available [62,63]. Importantly, Krüger et al. demonstrated *H. pylori*-induced motility and growth of *ex vivo *isolated gastric cells [63]. Access to primary cells is limited; hence it is important to investigate observed cellular molecular mechanisms *in vivo *as well. So far, it is still speculative if changes in cell morphology actually contribute to *H. pylori*-associated gastric diseases, even these processes likely influence host responses during decades of persistent *H. pylori *infections.

## *Helicobacter pylori *induced signal transduction pathways leading to a deregulated actin cytoskeleton independently of CagA

**Figure 3:**
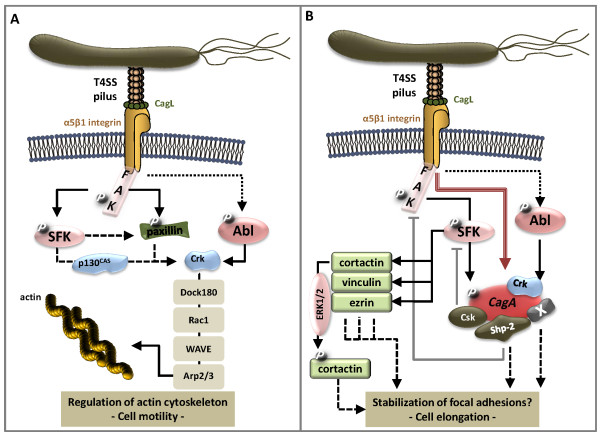
**Schematic overview of CagL and CagA-mediated signal transduction pathways involved in *H. pylori*-induced cell motility and elongation**. **(A) ***H. pylori *expresses CagL at the tip of the T4SS that directly binds to β1 integrins presented on gastric epithelial cells. Activated β1 integrin stimulates FAK and Src activity in early phases of *H. pylori *infections. FAK phosphorylates paxillin upon infection which might contribute to c-Abl-phosphorylated Crk signaling, which could be influenced by SFK activity *via *paxillin or p130^CAS^. FAK, SFKs and Abl kinase-mediated activation of Crk proteins can regulate the actin cytoskeleton through the DOCK180/Rac1/WAVE/Arp2/3 pathway contributing to epithelial cell migration. **(B) **CagL-integrin binding leads to the translocation of the *H. pylori *pathogenic factor CagA into the host cytoplasm. CagA is rapidly phosphorylated by kinases of the Src family (SFK) and bind to a large number of host cells factors (X) in its phosphorylated and non-phosphorylated form. Tyrosine phosphorylated CagA interacts with Shp-2 and Csk to inactivate FAK and Src in late phases of *H. pylori *infection. While inactivated Src is replaced by activated Abl kinases to maintain CagA phosphorylation, inactive Src leads to tyrosine dephosphorylation of Src target molecules ezrin, vinculin and cortactin. Cortactin is then serine phosphorylated by *H. pylori*-activated ERK1/2 kinases, which crucially contributes to cell elongation. Black arrows, *H. pylori*-induced direct signaling pathways. Dotted arrows, *H. pylori*-induced or Src-mediated indirect signaling pathways. Grey arrows, inactivating signaling pathways. Red arrow, CagA injection as the central step in the regulation of focal adhesions. P, phosphorylated proteins. X, host cell proteins.

While it is clear that *H. pylori *induces striking cytoskeletal changes in epithelial cells, knowledge of the signal transduction pathways is rare. In serum-starved cells, both CagA-positive and CagA-negative *H. pylori *strains mediated the formation of actin filaments and lamellipodial structures [64] implying activation of Rho GTPases. In fact, activation of Rac1 and Cdc42 has been demonstrated in *H. pylori*-infected AGS cells [65]. Microinjection of inactive Rac prevented actin cytoskeleton rearrangements in lamellipodial structures in *H. pylori*-colonized cells [64]. Through transfection of dominant-negative and catalytic-active cDNA constructs or using well-characterized GTPase-targeting toxins, Crk adapter proteins, Rac1 and H-Ras, but not RhoA or Cdc42 were identified as crucial components leading to *H. pylori*-induced cell elongation [66]. Consistent with actin polymerization in *H. pylori*-infected cells [64], activation of Rho GTPases occurs independently of CagA injection, but obviously required the T4SS apparatus [65]. Since CagL was identified as an adhesin for α5β1 integrins that is decorated at the tip of the T4SS allowing CagA injection and β1 integrin activation [24], it is tempting to speculate that CagL represents a promising candidate for stimulating Rho GTPase activation as well (Figure 3A). This hypothesis is currently supported by the finding that CagL-coated latex beads stimulated membrane ruffling *via *integrin-mediated activation of FAK and Src [24]. Another possible scenario proposes is OipA as an inducing factor since *oipA *mutants have been reported to less activate FAK presumably independently of *cag*PAI or CagA [67]; however experiments with recomplemented *oipA *mutants are pending.

The β1 integrin/FAK/Src pathway transmits signals to the actin cytoskeleton *via *paxillin, an important scaffolding protein located in focal adhesions [34]. In *H. pylori*-infected cells activated FAK phosphorylates tyrosine 118 in the paxillin protein (paxillin^Y118^) which was essential for cell motility in response to *H. pylori *[68]. Since phosphorylated paxillin^Y118 ^binds the adaptor protein v-crk sarcoma virus CT10 oncogene homolog (Crk) in response to cell adhesion, platelet-derived growth factor (PDGF) or angiotensin II [69], *H. pylori*-triggered paxillin^Y118 ^phosphorylation may also act upstream of the activation of Crk/DOCK180 (dedicator of cytokinesis)/Rac1/WAVE/Arp2/3 signal transduction pathway in *H. pylori*-infected cells, which has been detected in another study (Figure 3A) [70]. Alternatively, *H. pylori*-induced Src activity could activate p130^Cas ^leading to the recruitment of the Crk complex; however an involvement of p130^Cas ^in *H. pylori*-mediated cytoskeletal rearrangement still needs to be demonstrated (Figure 3A).

## Regulation of CagA-mediated host cell elongation

The *H. pylori*-induced changes in cell morphology are dominated by the drastic elongation of epithelial cells which involves active regulation of both the actin cytoskeleton and focal adhesions. Single-cell analyses suggested that *H. pylori*-dependent cell elongation might be mediated by deregulated focal adhesions rather than actin cytoskeleton rearrangement. Stabilized focal adhesions cause a defect in cell retraction leading to the formation of strong traction forces on motile *H. pylori*-infected cells [52]. CagA increases phosphorylation and subsequent activation of myosin light chain (MLC) in a Drosophila model [71]. The concomitant mispatterning of MLC results in cell elongation due to retraction failure and disruption of epithelial morphology and integrity. Based on a phospho-proteomic analysis the actin-binding protein vasodilator-stimulated phosphoprotein (VASP) was identified, which co-localized with focal adhesions of *H. pylori*-infected cells [72]. Down-regulation of VASP expression and inhibition of VASP phosphorylation blocked cell elongation in response to *H. pylori*, but it was not investigated whether phosphorylated VASP disturbed the disassembly of focal adhesions [72].

The significance of focal adhesions in promoting cell elongation has been emphasized by the finding that β1 integrin-mediated injection of CagA is important in the process of cell elongation [24]. Upon translocation, CagA localizes at the inner membrane of infected cells, where it is rapidly phosphorylated by the non-receptor tyrosine kinases c-Src, Fyn, Lyn and Yes of the Src family kinases (SFK) [73,74]. Phosphorylation sites were localized in a Glu-Pro-Ile-Tyr-Ala sequence (EPIYA motif), which exists as different 1-5 repeats, namely EPIYA-A, EPIYA-B, EPIYA-C in Western *H. pylori *isolates and EPIYA-A, EPIYA-B, EPIYA-D in East-Asian strains [75,76]. The Src-mediated CagA phosphorylation (CagA^PY^) is followed by a rapid inactivation of Src kinase activity, triggered by the binding of CagA to the C-terminal Src kinase (Csk) (Figure 3B) [54,58]. Src kinase inactivation then leads to the dephosphorylation of Src target proteins such as vinculin, ezrin and cortactin [49,54,77]. In fact, tyrosine phosphorylation of CagA^PY ^together with the dephosphorylation of SFKs and their target molecules are important in the process of regulation of the actin cytoskeleton and focal adhesions which contributes to the drastic morphological changes of *H. pylori*-infected cells (Figure 3B).

Another key molecule in *H. pylori*-stimulated cell elongation is Shp-2 (src homology 2 domain tyrosine phosphatase) (Figure 3B). Analysis of ectopically expressed CagA and isogenic phosphorylation-resistant mutants revealed that CagA^PY ^directly binds to Shp-2 which led to an increase of phosphatase activity of Shp-2 [78,79]. The CagA/Shp-2 complex has also been detected in the gastric mucosa of *H. pylori*-positive patients with gastritis and early stages of gastric cancer [80]. Activation of Shp-2 phosphatase activity has consequently been reported to inactivate FAK in cells that ectopically express CagA [81]. In contrast to activated FAK, dephosphorylated FAK cannot be localized in focal adhesions, which might support the development of the elongated cell phenotype. Contrary to this observation, CagL and OipA activate FAK in *H. pylori*-infected cells [24,67]. Recently, a new functional form of cortactin was reported, further underscoring the importance of cortactin as a critical mediator in signal transduction pathways in *H. pylori*-infected host cells (Figure 3B). After Src-mediated tyrosine dephosphorylation, cortactin becomes phosphorylated at serine 405 (cortactin^S405^). Phosphorylated cortactin^S405 ^strongly binds to and activates FAK. Cortactin^S405 ^phosphorylation was mediated by ERK1/2 kinases and might trap activated FAK leading to a disturbed turnover of focal adhesions (Figure 3B) [82]. This is one of the first identified mechanisms explaining why activation of mitogenic-activated protein (MAP) kinases via Rap1 GTPases [83] or protein kinases C (PKCs) [84] in response to *H. pylori *infections can contribute to cell elongation [61,70,82].

In contrast to dephosphorylated SFK target molecules, phosphorylation of CagA^PY ^is potently sustained by activated Abl kinases after inactivation of Src [60,85]. Abl kinases maintain CagA^PY ^phosphorylation and CagA^PY^-dependent downstream effects, which are still not fully understood. Interestingly, it was indicated that transfected East Asian-type CagA induced significantly stronger effects on rat cell growth than the Western CagA [86], which are obviously attributable to the different EPIYA motifs and their binding affinities to Shp-2 [75]. As it is not clear if Src and Abl kinases prefer different EPIYA motifs or exhibit similar phosphorylation affinities, further analyses are necessary to investigate the SFK and Abl kinase-mediated CagA phosphorylation.

Activated c-Abl consequently also phosphorylates Crk adapter proteins [60,85], which has been reported to interact with CagA^PY ^[70] linking a large CagA^PY ^recruited protein complex with signal transduction pathways towards the actin cytoskeleton (Figure 3B). Diverse, but coordinated signal transduction pathways converge on CagA^PY ^as an important central key molecule in *H. pylori*-mediated cell migration [76]. Beside Shp-2 as the first identified binding partner of CagA [78], many more binding partners for phosphorylated and non-phosphorylated CagA have been identified during the last years including Par1/MARK, c-Met, PLCγ (Phospholipase C gamma), ZO-1 (Zonula occludens-1), Csk (*c-Src tyrosine kinase*), Gab1 (*Grb-associated binder 1*), Crk (*CDC2-related protein kinase*) proteins, Grb2 and the cell adhesion protein E-cadherin [10,33]. It is still unclear whether one CagA molecule can bind to more than one interaction partner simultaneously. But for most of these identified binding proteins it could be shown that they play a role in the induction of the *H. pylori*-dependent scatter phenotype.

## Conclusions

Infection of gastric epithelial cells with *H. pylori in vitro *induces a strong motility response; however, our current understanding of the complex molecular mechanism contributing to this phenotype is still rudimentary understood. Although data are steadily increasing indicating that α5β1 integrin/CagA signaling is involved in stabilization of focal adhesion at the rear of the motile cell, it is unclear how these processes can be differentiated from the cellular mechanisms stimulating the assembly of nascent focal adhesions and rearrangement of the actin cytoskeleton at the leading edge. Hence, further studies are necessary to investigate signal transduction pathways controlling these locally demarcated regions in *H. pylori *infected host cells *in vitro *as well as *in vivo*, which might have consequences on the physiological balance and integrity of the gastric epithelium *in vivo*.

## Competing interests

The authors declare that they have no competing interests.

## Authors' contributions

SW, MG and GR wrote the manuscript. All authors read and approved the final manuscript.
